# Anti-inflammatory Nanomedicine for Cardiovascular Disease

**DOI:** 10.3389/fcvm.2017.00087

**Published:** 2017-12-22

**Authors:** Shunsuke Katsuki, Tetsuya Matoba, Jun-ichiro Koga, Kaku Nakano, Kensuke Egashira

**Affiliations:** ^1^Department of Cardiovascular Medicine, Kyushu University Graduate School of Medical Sciences, Fukuoka, Japan; ^2^Center for Excellence in Vascular Biology, Division of Cardiovascular Medicine, Department of Medicine, Brigham and Women’s Hospital, Harvard Medical School, Boston, MA, United States; ^3^Department of Cardiovascular Research, Development, and Translational Medicine, Center for Cardiovascular Disruptive Innovation, Kyushu University, Fukuoka, Japan

**Keywords:** coronary artery disease, inflammation, nanomedicine, monocytes, macrophages

## Abstract

Coronary artery disease, in the development of which inflammation mediated by innate immune cells plays a critical role, is one of the leading causes of death worldwide. The 3-hydroxy-3-methylglutaryl coenzyme A reductase inhibitors (statins) are a widely used lipid-lowering drug that has lipid-independent vasculoprotective effects, such as improvement of endothelial dysfunction, antioxidant properties, and inhibitory effects on inflammation. Despite recent advances in lipid-lowering therapy, clinical trials of statins suggest that anti-inflammatory therapy beyond lipid-lowering therapy is indispensible to further reduce cardiovascular events. One possible therapeutic option to the residual risk is to directly intervene in the inflammatory process by utilizing a nanotechnology-based drug delivery system (nano-DDS). Various nano-sized materials are currently developed as DDS, including micelles, liposomes, polymeric nanoparticles, dendrimers, carbon nanotubes, and metallic nanoparticles. The application of nano-DDS to coronary artery disease is a feasible strategy since the inflammatory milieu enhances incorporation of nano-sized materials into mononuclear phagocytic system and permeability of target lesions, which confers nano-DDS on “passive-targeting” property. Recently, we have developed a polymeric nanoparticle-incorporating statin to maximize its anti-inflammatory property. This statin nanoparticle has been tested in various disease models, including plaque destabilization and rupture, myocardial ischemia-reperfusion injury, and ventricular remodeling after acute myocardial infarction, and its clinical application is in progress. In this review, we present current development of DDS and future perspective on the application of anti-inflammatory nanomedicine to treat life-threatening cardiovascular diseases.

## Anti-Inflammatory Therapeutics for Coronary Artery Disease

Coronary artery disease is the leading cause of death worldwide and can be life threatening especially when it develops into acute myocardial infarction (AMI), the most severe type of atherosclerotic cardiovascular disease. The 3-hydroxy-3-methylglutaryl coenzyme A (HMG-CoA) reductase inhibitors (statins) are potent inhibitors of cholesterol synthesis and established therapies for the prevention of coronary artery disease. The first randomized trial to demonstrate that cholesterol-lowering therapy with statins improves prognosis in patients with high cholesterol and coronary artery disease, the Scandinavian Simvastatin Survival Study (4S), was reported in 1994 (1). Since then, lipid-lowering therapy with statins has become the mainstay for the prevention of coronary artery disease. The subsequent studies for the secondary prevention such as the CARE (Cholesterol and Recurrent Events) (2) and LIPID (Long Term Intervention with Pravastatin in Ischemic Disease) trials (3) further extended that the benefits of statins to the majority of patients whose cholesterol levels were in the normal range. The WOSCOPS (West of Scotland Coronary Prevention Study) (4) and the AFCAPS/TexCAPS (Air Force/Texas Coronary Atherosclerosis Prevention Study) (5) also extended the benefits for the primary prevention of atherosclerotic cardiovascular diseases. A decade after 4S, Ridker et al. demonstrated in the JUPITER (Justification for the Use of Statins in Primary Prevention: an Intervention Trial Evaluating Rosuvastatin) trial that high-intensity cholesterol-lowering therapy with statins reduced high-sensitivity C-reactive protein levels to lower than 2 mg/L and then achieved further risk reduction of cardiovascular events even among patients with normal cholesterol levels (6). Statins have a wide range of lipid-independent cardiovascular protective effects (so called “pleiotropic effects”), such as improvement of endothelial dysfunction, antioxidant properties, and inhibitory effects on inflammation (7) This landmark study suggested that anti-inflammatory therapy beyond lipid-lowering therapy is needed to further reduce cardiovascular events. At present, plaque erosion appears on the rise as a cause of acute coronary syndrome (ACS) in this statin era (8), but plaque rupture of unstable plaques with macrophage accumulation, fibrous cap thinning, and lipid deposition, followed by arterial occlusive thrombosis remains the primary mechanisms of ACS. Despite recent advances in lipid-lowering therapy by the emergence of Niemann-Pick C1-Like 1 inhibitor (ezetimibe) (9, 10) and proprotein convertase subtilisin/kexin type 9 inhibitors (11), there are still residual risks of cardiovascular events. One possible approach to the residual risks is to directly intervene in the inflammatory process during atherogenesis. Current medicinal therapy, including statins, has limitation of bioavailability in the diseased organs. Most small molecule drugs are absorbed from the intestines, metabolized in the liver, delivered to the bloodstream, and excreted from the kidneys, that is, drugs need to overcome the physiological barriers to achieve effective concentration in the blood and tissue. Adverse effects that any drugs possess may also limit their maximum dose in terms of safety. Recent innovation in nanomedicine can achieve effective drug delivery to targeted organs and cells, and spare undesirable adverse effects. In the first part of this review, we summarize recent advances in nanotechnology-based drug delivery system (nano-DDS). In the second part, we demonstrate the data of nano-DDS of statins in coronary artery disease for maximizing its anti-inflammatory effects.

## Various Nano-Sized Materials as Drug Delivery Systems

Currently, various nano-sized materials (nanomaterials) have been tested and approved in clinical settings: lipid nanoparticles such as micelles or liposomes (12), polymeric nanoparticles (13), dendrimers (14), carbon nanotubes, and metallic nanoparticles. The characteristics of these materials and clinical applications are as follows (Table 1).

**Table 1:** Characteristics of various nano-sized materials as drug delivery systems.

	Micelle	Liposome	Polymer nanoparticle	Dendrimer	Carbon nanotube	Metallic nanoparticle
Geometry	Spherical vesicles composed of a monolayer of lipids or synthetic amphiphiles	Spherical vesicles composed of phospholipids bilayers	An assembly of macromolecular polymers	Highly branched monodisperse macromolecules from a central core	Single or multi-walled tubular form of graphite sheets	A magnetic core coated with hydrophilic polymers
Size (nm)	10–100	40–1,000	20–1,000	3–20	0.5–3.0 × 20–1,000	60–150 (core 4–5)
Features	Encapsulation of hydrophobic agents inside	Encapsulation of hydrophilic agents inside, embedding hydrophobic agents in the membrane	Incorporation of hydrophilic and hydrophobic agents, controlled release of incorporated agents	Multivalent properties by exterior funciton groups	Encapsulation of agents into its inner space with chemically modified external surface	Contrast agents for biological imaging, photothermal properties

### Micelles

Micelles consist of lipids and other amphiphilic artificial molecules, such as polymers. Micelles are self-assembling in aqueous solution to form a monolayer with a hydrophobic phase inside so that they can incorporate hydrophobic therapeutic agents. The diameter of micelles is usually 10–100 nm and the enclosed space is more confined than that of liposomes.

### Liposomes

Since United States Food and Drug Administration (FDA) approved liposomal formulations of doxorubicin and amphotericin B in the mid-1990s (15), liposomes have been investigated most extensively in nanomedicine with approval due to less toxicity for *in vivo* application and capacity to deliver a variety of payloads. Liposomes mainly consist of phospholipids to form bilayers with an aqueous phase inside, which confer superior biocompatibility on liposomes. They can not only incorporate hydrophilic therapeutic agents inside but also hydrophobic agents in the liposomal membrane. Macromolecular drugs, including nucleic acid and crystalline metals, can be also incorporated in liposomes. The diameter of liposomes is usually 40–1,000 nm. Liposomes obtain specific characteristics through modification of its surface with polymers, antibodies, and protein. PEGylated liposomal doxorubicin (Doxil^®^) is the first FDA-approved nanomedicine and enhanced the drug concentration in malignant effusions by 4-fold to 16-fold, while reducing cardiotoxic side effects (16).

### Polymeric Nanoparticle

Food and Drug Administration-approved polymers, polylactic acid (PLA), polyglycolic acid (PGA), and poly lactic-co-glycolic acid (PLGA), are widely used for the synthesis of polymeric biodegradable nano-DDS, because they are eliminated from the body in the form of water and carbon dioxide. PLGA is a copolymer of PLA and PGA, the most frequently used constitution among these polymers, and is being tested for a DDS for intractable diseases, including cardiovascular disease. PLA is more hydrophobic, whereas PGA is more hydrophilic; the degradation speed of PLGA can be adjusted by the PLA:PGA ratio and their molecular weights, which achieves controlled release of incorporated drugs. PLGA polymers incorporate hydrophilic and hydrophobic therapeutic agents, including chemicals and nucleotides by emulsion solvent diffusion methods. The diameter of polymeric nanoparticles widely ranges from 20 to 1,000 nm. FDA-approved PLGA nanoparticle utilizing these advantages is leuprolide acetate (a testosterone inhibitor)-incorporated nanoparticle for prostate cancer (Eligard^®^). PLGA achieves slow and sustained leuprolide acetate release after subcutaneous injection.

### Dendrimer

Dendrimers are dendritically expanded macromolecules with monodisperse structure that consist of a central core, branching interior, and exterior functional groups (14). Dendrimers can incorporate therapeutic agents in their three-dimensional branching interior voids and work as a drug delivery carrier. The number of repeating branching cycles is called generations. Exterior function groups increase exponentially as generation increases, which confer multivalent properties on dendrimers. This multivalent effect has the advantage of enhancing the binding capacity when its exterior surface is modified with some ligands or antibodies as an active targeting (17).

### Carbon Nanotube

Carbon nanotubes are a subfamily of fullerenes and consist of graphite sheets rolled up into tubular form. As drug carriers, they can incorporate drugs into its inner space and have a chemically modified external surface with biomolecules, such as proteins and nucleotides, for selective targeting. Carbon nanotube-based anticancer therapy such as cisplatin-incorporated carbon nanohorn is currently being investigated (18).

### Metallic Nanoparticle

Metallic nanoparticles include iron oxide and gold nanoparticles. Iron oxide nanoparticles consist of a magnetic core (4–5 nm) and hydrophilic polymers such as dextran or poly(ethyleneglycol)s. Superparamagnetic iron oxide (SPIO) (60–150 nm) has been investigated as a contrast agent for magnetic resonance imaging (MRI). Ferumoxytol (Feraheme^®^), caraboxymethydextran-coated iron oxide, is the only FDA-approved SPIO. The clinical use of ferumoxytol is limited to iron replacement therapy for patients with chronic kidney disease and is under investigation as an imaging agent. Resovist^®^, carboxydextran-coated iron oxide, has been withdrawn from FDA-approved drugs due to lack of clinical users, and is currently available in limited countries including Japan (19). Gold nanoparticles has unique photothermal properties, tunable size and shape, and easily modified surface. No gold nanoparticles have been clinically approved to date, but they are actively investigated especially in research fields targeting cancer (20).

Although micro-sized particles are considered to be biologically inert, nano-sized materials could activate innate immune sensors. In fact, nano-sized inorganic metal oxides, such as silica dioxide (SiO_2_) and titanium dioxide (TiO_2_) (21), and silver nanoparticle (22) have been reported to activate the NLR pyrin domain containing 3 (NLRP3) inflammasome in human macrophages. Since carbon nanotubes are fiber-shaped material, the morphology of which is similar to that of asbestos, the concern for the toxicity of carbon nanotubes has been raised (23). In animal models, intratracheal exposure with the above-mentioned nanomaterials demonstrated airway inflammation (21, 24). The activation of inflammasome is mediated by potassium efflux (25), lysosome degradation that results in cathepsin B leakage, generation of reactive oxygen species (ROS) (26), and production of adenosine (27). These data suggested that greater caution might be needed to these widely produced nanomaterials.

## *In Vivo* Kinetics of Nanoparticle-Mediated Drug Delivery System

**Figure 1:**
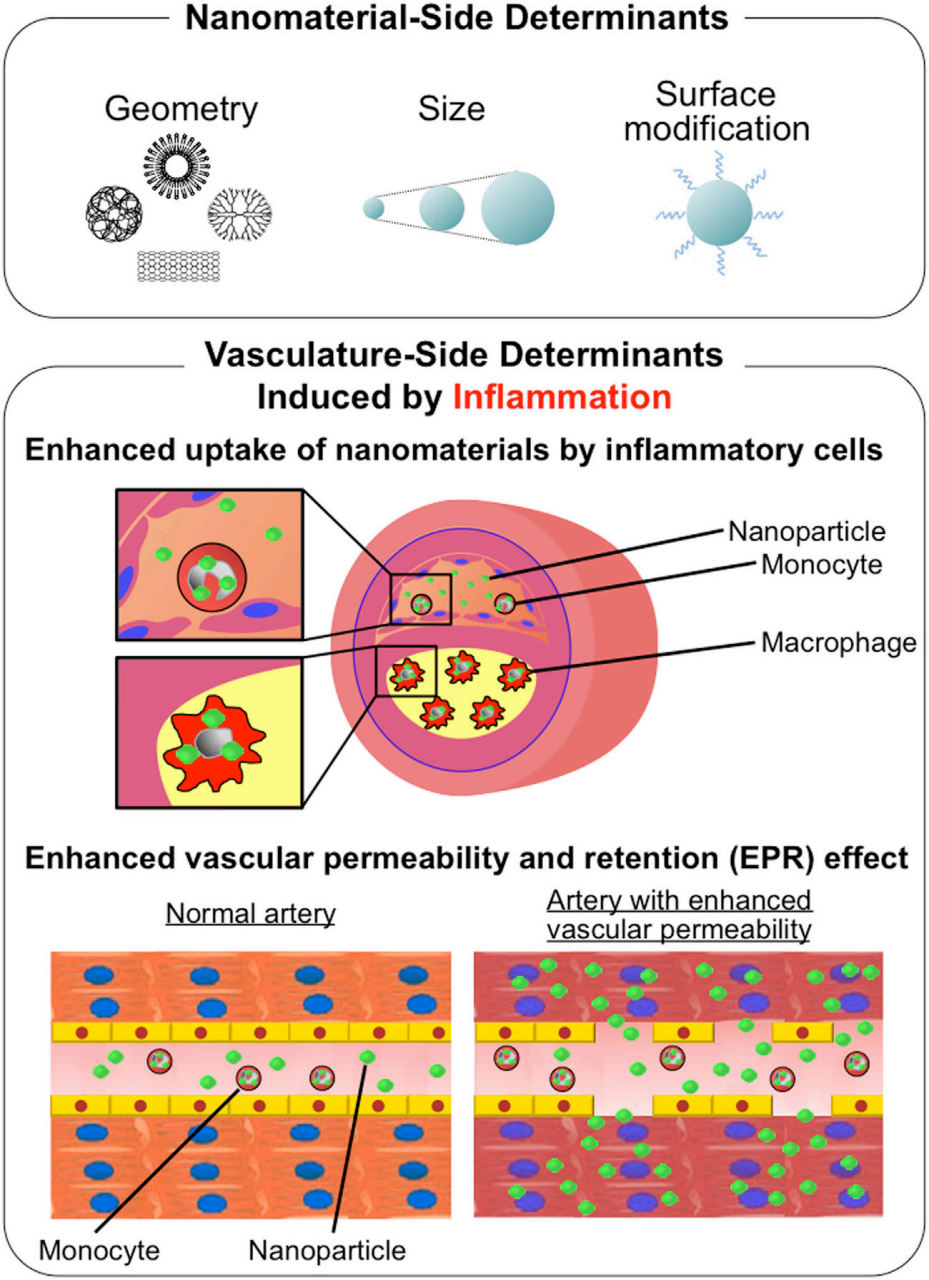
Schematic description of determinants of physiological behavior of nanomaterials. (Upper panel) Nanomaterial-side determinants: geometry, size, and surface modification. (Lower panel) Vasculature-side determinants induced by inflammation: enhanced uptake of nanomaterials by inflammatory cells and enhanced vascular permeability and retention (EPR) effect.

Although there are numerous determinants to affect *in vivo* kinetics of nano-DDS other than geometry as previously discussed, the size and surface modification of nanoparticles are the most important in the physiological behaviors of nano-DDS. Large-sized nanomaterials (>1,000 nm in diameter) tend to accumulate in the liver and lungs, and sometimes can be the cause of microemboli in capillaries, while small-sized nanomaterials (<10 nm) tend to be excreted from the kidneys. Middle-sized nanomaterials (10–1,000 nm) remain in circulation for longer time avoiding renal excretion. These circulating nanomaterials are generally incorporated by the mononuclear phagocytic system (MPS) in the liver, spleen, lymph nodes, and bone marrow (28). A surface modification with polyethylene glycol (PEG) serves as a hydrophilic shield that reduces protein absorption and undesirable non-specific interaction with MPS, which is preferable for nano-DDS in cancer. However, incorporation of nanomaterials into MPS itself is one of the intended mechanisms of drug delivery, especially when targeting inflammatory diseases including atherosclerosis. On the other hand, accumulation of nanomaterials depends on the permeability of target lesions. In tumor blood vessels, inflammatory atherosclerotic lesions, and ischemic myocardium, nanomaterials extravasate from blood vessels due to enhanced permeability. Tumors lack functional lymphatic vessels in their tumor microenvironment, which enhances the accumulation of nanomaterials. These phenomena are referred to as “enhanced permeability and retention effects” (29) or “passive-targeting” (Figure 1).

By contrast, “active-targeting” strategy employs target-specific structures, such as antibodies and proteins on nanomaterials, which bind to the target molecule that is specific for a certain disease process. “Active-targeting” strategy is being developed in cancer therapeutics targeting molecules associated with angiogenesis and cell proliferation (30). Adhesion molecules are one of the candidates for cardiovascular imaging as described later in this review, and innovative cardiovascular imaging using “active-targeting” strategy is being investigated. Regardless of the benefits of “active targeting,” only a few clinically validated nanomaterials utilize this strategy. Engineered protein combining IL-2 and diphtheria toxin (Ontak^®^) is the only FDA-approved “active-targeting” nanomaterial to date (15).

## The Role of Monocytes/Macrophages in Coronary Artery Disease

Monocytes/macrophages play a key role in the inflammatory hypothesis of atherothrombosis (31). Atherogenesis starts with endothelial dysfunction caused by oxidative, hemodynamic, or biochemical stimuli (from smoking, hypertension, or dyslipidemia) and inflammatory factors. Endothelial dysfunction leads to enhanced permeability for cholesterol-containing low-density lipoprotein (LDL) particles and the expression of adhesion molecules to promote adhesion of circulating monocytes. Adhesive monocytes migrate into subendothelial space by chemoattractant proteins including monocyte chemoattractant protein-1 (MCP-1), where monocytes are differentiated into macrophages by macrophage-colony stimulating factor and activated through phagocytosis of oxidized lipid components to become foam cells. Activated macrophages secrete inflammatory cytokines to trigger positive feedback loop between cytokines and immune cells and matrix metalloproteinases (MMPs) leading to fibrous cap thinning. Rupture-prone plaques are characterized by the abundant accumulation of innate immune cells (mainly monocytes/macrophages), lipid core formation, and induction of several proteinases that catabolize the extracellular matrix (32).

Monocytes are functionally heterogeneous and are classified into at least two major subsets in mice: inflammatory monocytes (Ly-6C^high^CCR2^+^CX3CR1^low^) and non-inflammatory monocytes (Ly-6C^low^CCR2^−^CX3CR1^high^) (33). Previously, we developed a mouse model of “plaque rupture” utilizing a high-fat diet and angiotensin II infusion in apolipoprotein E (ApoE)-deficient mice and reported that adoptive transfer of Ly-6C^high^CCR2^+^ inflammatory monocytes increases buried fibrous caps in the brachiocephalic arteries (34), suggesting a critical role for inflammatory monocytes in plaque destabilization. Although their functional similarities have not been fully determined, CD14^++^CD16^−^ classical monocytes and CD14^+^CD16^++^ non-classical monocytes are described as their respective counterparts of Ly-6C^high^ and Ly-6C^low^ monocytes according to their chemokine receptor expression in humans, and intermediate monocytes (CD14^++^CD16^+^) have been recently identified as not only a transitory state but also likely to possess unique features (35). There were not any significant differences in the peak levels of circulating CD14^++^CD16^−^ monocytes among the patients with AMI, unstable angina pectoris (UAP), and stable angina pectoris (SAP), but that of circulating CD14^+^CD16^+^ monocytes (defined as CD14^++^CD16^+^ plus CD14^+^CD16^++^ monocytes) were significantly decreased in AMI patients compared with those in patients with UAP or SAP (36). A conventional paradigm proposed two subpopulations in macrophages corresponding to the monocyte heterogeneity: “classically activated or inflammatory M1 macrophages” and “alternatively activated or anti-inflammatory M2 macrophages.” Although the balance between “M1” and “M2” subpopulations may contribute to the development of cardiovascular disease, emerging evidence suggests that heterogeneity of macrophage subpopulation seems much more complex than “M1” and “M2” dichotomy (37). “M2” macrophages are divided into at least three subpopulations according to the stimulus they are activated; “M2a,” “M2b,” and “M2c.” All “M2” macrophages have anti-inflammatory properties to secrete IL-10 and TGF-β. “M2b” is an exception because they additionally secrete pro-inflammatory cytokines, such as IL-1β, IL-6, and TNFα. The tissue microenvironment in atherosclerotic plaques can affect macrophage population (38). Oxidized phospholipids induce “Mox” and intraplaque hemorrhage induces “Mhem (HA-mac)” or “M(Hb).” Although localization of “Mox” in human atherosclerotic lesions remains to be elucidated, the population was detected in approximately 30% of macrophages in advanced atherosclerotic plaque of LDL receptor-deficient mice. “Mox” not only shows an pro-inflammatory phenotype by producing cyclooxygenase-2 and IL-1β but also control redox status by inducing redox-regulating genes such as heme oxygenase-1 (HO-1), sufiredoxin-1 (Srnx1), and thioredoxin reductase 1 *via* nuclear factor erythroid 2-like 2 (NFE2L2). They also display reduced chemotactic and phagocytic capacities, which might contribute to perpetuation of inflammation and tissue damage (39). “Mhem” (previously named as “HA-mac” by the same investigators), originally defined as CD163^high^ human leukocyte antigen-DR^low^, was detected in human hemorrhaged atherosclerotic plaque. “Mhem” is an atheroprotective subpopulation that drives adaptation to intraplaque hemorrhage through activation transcription factor 1-mediated induction of HO-1 by heme (40). Another investigators named this population “M(Hb)” and further demonstrated that “M(Hb)” has high expression of “M2 markers” mannose receptor and CD163, and reduces ROS production and promotes LXRα-mediated reverse cholesterol transport (41). Platelet chemokine CXCL4 induces “M4” macrophages. They lack CD163 and are unable to increase HO-1 production in response to hemoglobin or hemoglobin–haptoglobin complexes. Co-expression of MMP7 and S100 calcium-binding protein A8 (S100A8) in “M4” in human atherosclerotic plaques might lead to plaque destabilization by increased expression of MMP7 and affect lesion progression through decreased macrophage proliferation (42). In this way, the description of macrophage activation has been expanded and confusing, and then a group of scientists proposed the updated nomenclature to define the activator, i.e., M(IFNγ), M(LPS), M(IL-4), and M(IL-10), instead of the current complex nomenclature in *in vitro* experiments. This helps researchers to avoid using different definitions of activated macrophages. They also provide the framework within which researchers place a given population for *in vivo* experiments (37).

Macrophage is also a protagonist in myocardial ischemia-reperfusion (IR) injury and wound healing process of ischemic heart disease following AMI. In patients with ST-segment elevation acute MI (STEMI), early reperfusion is a standard therapy to salvage viable myocardium and limit MI size. Regardless of significant reductions in door-to-balloon time in the last decade, the mortality of patients with MI has not improved as shown in recent cohort studies. It is well recognized that the reperfusion of coronary arteries can paradoxically induce cardiomyocyte death, called “myocardial IR injury.” The major contributing factors in myocardial IR include ROS, calcium overload, and the rapid restoration of physiological PH at reperfusion, which induces the opening of the mitochondrial permeability transition pore that leads to the necrosis and apoptosis of cardiomyocytes in the first several minutes of reperfusion. Over several hours after reperfusion, myocardial inflammation contributes to cardiomyocyte apoptosis and the healing of infarcted myocardium (43, 44). The recruitment of neutrophils and inflammatory monocytes is an established phenomenon after myocardial injury. Uncontrolled macrophage infiltration following MI results in ischemic heart failure. We and another group demonstrated that infarcted myocardium sequentially recruits Ly-6C^hi^ monocytes and Ly-6C^lo^ monocytes in murine MI model (45) and IR injury model (46); Ly-6C^hi^ monocyte dominates in early phase to exhibit inflammatory functions including phagocytic and proteolytic activity, while Ly-6C^low^ monocyte dominates in later phase to resolute inflammation and promote angiogenesis. In consistent with this preclinical data, circulating CD14^++^CD16^−^ and CD14^+^CD16^+^ monocytes sequentially increase in patients with AMI. The peak levels of circulating CD14^++^CD16^−^ monocytes were negatively correlated with the degree of myocardial salvage, suggesting that manipulating classical inflammatory monocyte could be a therapeutic target for salvaging for ischemic myocardial damage after MI (36).

Monocyte chemoattractant protein-1 belongs to the CC chemokine subfamily and its primary receptor CCR2 is highly expressed on a subpopulation of monocytes (Ly-6C^high^ monocytes in mice and CD14^++^CD16^−^ monocytes in humans). These inflammatory monocytes depend on CCR2 to migrate into the injured tissue. We previously demonstrated that systemic gene therapy with plasmids encoding 7ND, a deletion mutant of MCP-1, limits atherogenesis associated with increased lesional extracellular matrix content, one of characteristics of stable plaques, without any effects on serum lipid concentration (47). Furthermore, this gene therapy not only limits progression of established preexisting atheroma but also leads to plaque stabilization (48). We then utilized the 7ND-incorporated PLGA nanoparticle for interfering with MCP-1/CCR2 signaling and demonstrated inhibition of macrophage accumulation in the atherosclerotic plaque, followed by amelioration of morphological characteristics similar to human destabilized/ruptured plaque in the brachiocephalic artery of ApoE-deficient mice (34). Leuschner et al. demonstrated that inhibition of monocyte CCR2 with siCCR2-incorporated lipid nanoparticle inhibits macrophage accumulation in sites of inflammation and reduces atherosclerotic formation and infarct size in myocardial IR injury (49). Another group reported that blockade of CCR2 markedly reduced macrophage infiltration in ischemic lesions that results in attenuation of myocardial IR injury in mice *via* inhibition of macrophage-related oxidative stress and MMPs (50) They also demonstrated that macrophage infiltration into infarcted tissue was impaired in CCR2^−/−^ mice after coronary ligation and the CCR2 deficiency ameliorates post-MI left ventricular (LV) remodeling *via* inhibition of macrophage-related MMPs (51). These data suggested that inflammatory subpopulations of monocytes/macrophages are feasible therapeutic targets for coronary artery disease.

Other immune cells, including dendritic cells (DCs), T cells, and B cells, also play important roles in atherosclerosis. A predominant subpopulation of T cells in atherosclerotic lesion is CD4^+^ T cells. CD8^+^ T cells and natural killer T cells are minor T cell subpopulations. Antigen-presenting cells such as macrophages and DCs process the disease-related antigens including oxidized LDL, heat-shock proteins and microbes, and present them to CD4^+^ T cells as fragments loaded onto major-histocompatibility-complex class II molecules, which translates innate to adaptive immunity and is one of the therapeutic targets in adaptive immunity. In fact, vaccination targeting oxidized LDL-immuned DCs ameliorates atherosclerosis in LDL receptor-deficient mice (52). Activated T cells subsequently produce the type 1 helper T (Th1) cytokines (e.g., interferon γ) to initiate pro-inflammatory responses. Atherosclerotic lesions usually contain Th1 cytokines rather than the type 2 helper T (Th2) cytokines. In contrast, regulatory T cells modulate the process by producing anti-inflammatory cytokines, such as IL-10 and transforming growth factor-β (TGF-β) (53). The role of B cells in the progression of atherosclerosis still remains poorly understood, but it has recently gained more attention. B cells have two main subsets in mice based on their origin; B1 and B2. The role of the majority subset B2 cells in atherosclerotic lesion is controversial, while B1 cells seem to be atheroprotective (54). The corresponding B cell subsets in humans have not been clearly demonstrated. Nanomedicine can be applied to those atherosclerosis-regulating immune cells. However, the application of nanomedicine to atherosclerotic disease is extremely limited so far. A previous study demonstrated that intranasal immunization with chitosan/pCETP nanoparticles inhibits atherosclerosis in rabbit, in which the nanoparticulation enabled an efficient delivery of the antigen peptide to antigen presenting cells while sparing mucociliary clearance (55). Further studies are needed for the development of nanomedicine targeting adaptive immunity by utilizing nanoparticles as antigen delivery carriers of vaccination.

## Nanomaterials for Imaging of Macrophages

We have described that macrophage-mediated inflammation plays a crucial role in coronary artery disease. Hence, imaging of macrophages provides insight for future therapeutic options for cardiovascular diseases, in which several nanomaterials are being investigated as new imaging modalities. Developing those modalities for imaging macrophages is also indispensable in terms of quantitative analysis of therapeutic effects of anti-inflammatory drugs. Computed tomography (CT) is widely used for imaging of coronary artery in clinical setting. Nanomaterials for CT are limited because it requires high concentrations of absorbent nanomaterials to detect macrophages with X-ray. N1177, a crystalline iodined aroyloxy ester covered with a polymer, can detect macrophage-rich arterial walls in rabbits by CT (56). Macrophage-targeted gold high-density lipoprotein (HDL) nanoparticle can be localized in the macrophages of atherosclerotic plaque in the aorta of ApoE-deficient mice (57). MRI has high soft tissue contrast resolution and can detect plaque morphology non-invasively without radiation exposure. SPIO nanoparticle and ultrasmall superparamagnetic iron oxide (USPIO) are nanomaterial developed as the negative contrast for MRI. SPIO- and USPIO-based contrast agents consist of iron oxide core with hydrophilic polymeric coating, such as dextran-coated monocrystalline iron oxide nanoparticle-47 (MION-47) and dextran-crosslinked iron oxides. High-resolution MRI after administration of MION-47 can assess macrophage burden in atheromata induced by balloon injury of cholesterol-fed New Zealand White rabbits (58). In clinical setting, ATHEROMA trial has been conducted to examine USPIO-related signal change in patients with carotid stenosis >40%, but USPIO-enhanced MRI did not predict cardiovascular events significantly (59). On the other hand, USPIO-based contrast, ferumoxytol, has succeeded in characterizing infarcted myocardium mainly by detecting infiltrating macrophages (60). There are some reagents that adopt “active-targeting” strategy to image lesional macrophages. One good example is that iron oxide nanoparticle conjugated with ligand of vascular cell adhesion molecule 1 (VCAM-1) visualized VCAM-1-expressing endothelial cells and macrophages in ApoE-deficient mice (61). Nano-sized probes for near infrared fluorescence (NIRF) are used in animal studies. The excitation and emission wavelengths of NIRF probes range from 600 to 900 nm. In that range, the absorbance and scattering of biological tissues are relatively low. These probes are designed to be activated when target protease cleaves protease-specific peptide substrates linked to quenched fluorescent dyes. MMP (62, 63) and cathepsin are used as the substrates to image macrophage burden (64).

## Anti-Inflammatory Therapeutics with Nanomaterials for Coronary Artery Disease

### Atherosclerosis

**Figure 2:**
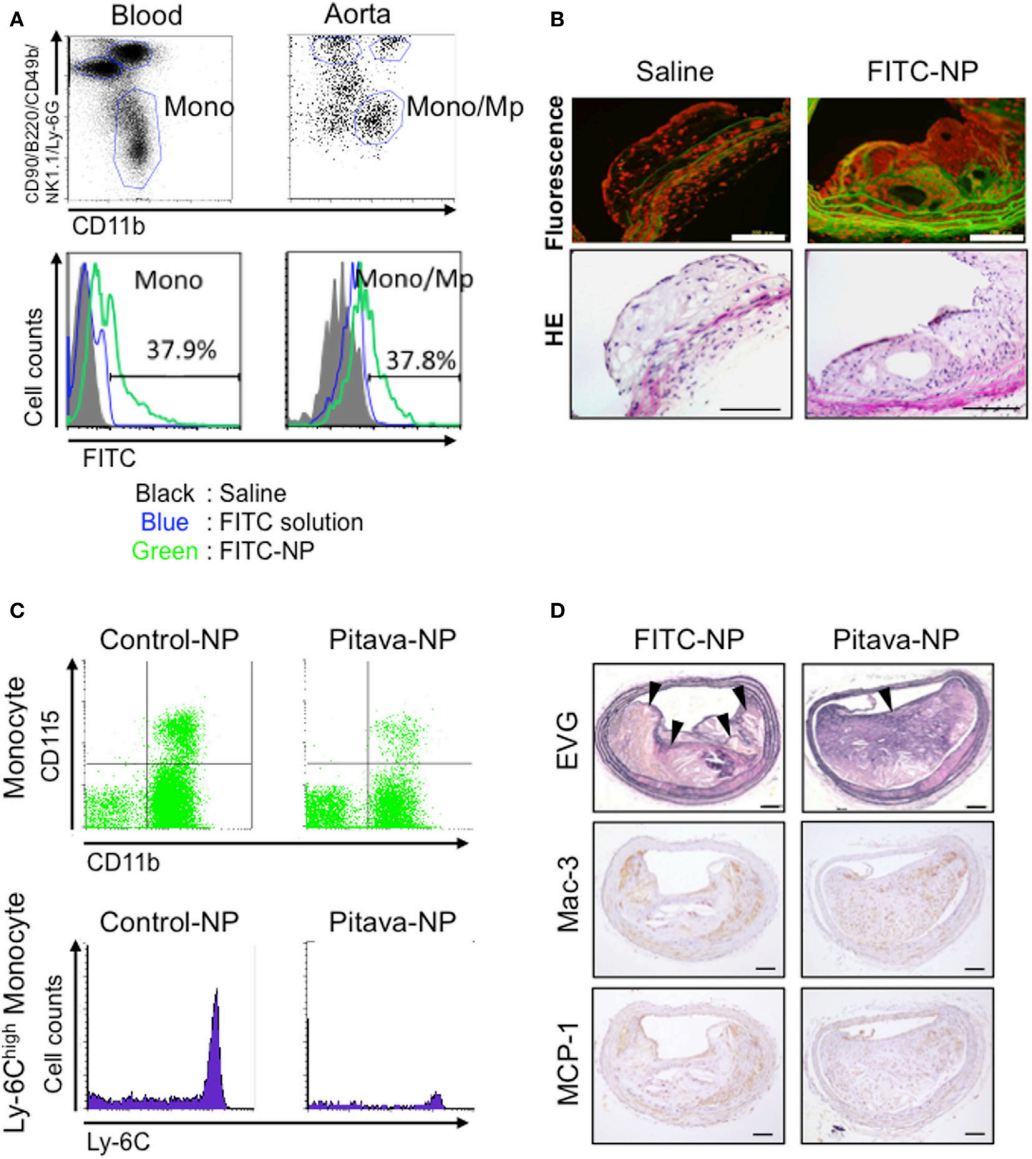
Efficacy of pitavastatin-nanoparticle in atherosclerotic mice. **(A)** Flow cytometry of circulating leukocytes 2 days after intravenous injection of poly lactic-co-glycolic acid nanoparticles encapsulated with FITC (FITC-NP). The histograms demonstrate FITC uptake by monocytes in the blood and the aorta. **(B)** Fluorescent and light micrographs of brachiocephalic arteries 24 h after intravenous injection of saline or FITC-NP. **(C)** Flow cytometric dot plots and histograms of leukocytes from mice injected intravenously with control (empty)-NPs or pitavastatin-NPs (Pitava-NP). **(D)** Weekly intravenous injection of Pitava-NP increased fibrous cap thickness and decreased the number of buried fibrous caps in the atherosclerotic plaques with reduced macrophage accumulation (Mac-3) and monocyte chemoattractant protein-1 (MCP-1) expression. Arrows indicate disrupted/buried fibrous caps. We reused these data according to the Copyright Transfer Agreement with the publisher (34, 66).

We have recently developed an innovative nano-DDS utilizing polymeric PLGA nanoparticle-incorporating pitavastatin (Pitava-NP) without PEGylation to enhance the anti-inflammatory effects of statin on monocyte/macrophage-mediated inflammation of coronary artery disease. The average diameter of polymeric nanoparticles is 200 nm. Fluorescence-labeled nanoparticle (FITC-NP) was incorporated mainly Lineage (CD90/B220/CD49b/NK1.1/Ly-6G)^−^CD11b^+^ monocytes in blood and Lineage^−^CD11b^+^ monocytes/macrophages in aorta by intravenous injection (Figure 2A). Fluorescence microscopic images demonstrated that FITC signal was observed in atherosclerotic plaque of brachiocephalic artery 24 h after intravenous injection of FITC-NP, suggesting that FITC-NP was passively delivered to atherosclerotic lesion with enhanced permeability (Figure 2B). Time course analysis of FITC signal in peripheral and aortic leukocytes by flow cytometry revealed that the delivery of FITC-NP to peripheral monocytes was followed by its delivery to aortic macrophages over 2–7 days after injection, suggesting a direct delivery of intravenous PLGA nanoparticles to blood monocytes, which gradually migrate to the atherosclerotic aorta. Weekly intravenous treatment with Pitava-NPs reduced circulating inflammatory Ly-6C^high^ monocytes, macrophage accumulation in the atherosclerotic lesions of the aortic root, and ameliorated morphological characteristics similar to human destabilized/ruptured plaque in the brachiocephalic arteries of ApoE-deficient mice (Figures 2C,D) (34). In consistent with these data, a preclinical study from other investigators reported that a statin-loaded reconstituted HDL (rHDL) nanoparticle inhibits atherosclerotic formation. Using dual gadolinium and fluorescent dye-labeled rHDL nanoparticle, they demonstrated that intravenously administered rHDL was incorporated into lesional monocytes and macrophages, and inhibits plaque formation with reduced macrophage content in the aortic root (65).

### Myocardial IR Injury

**Figure 3:**
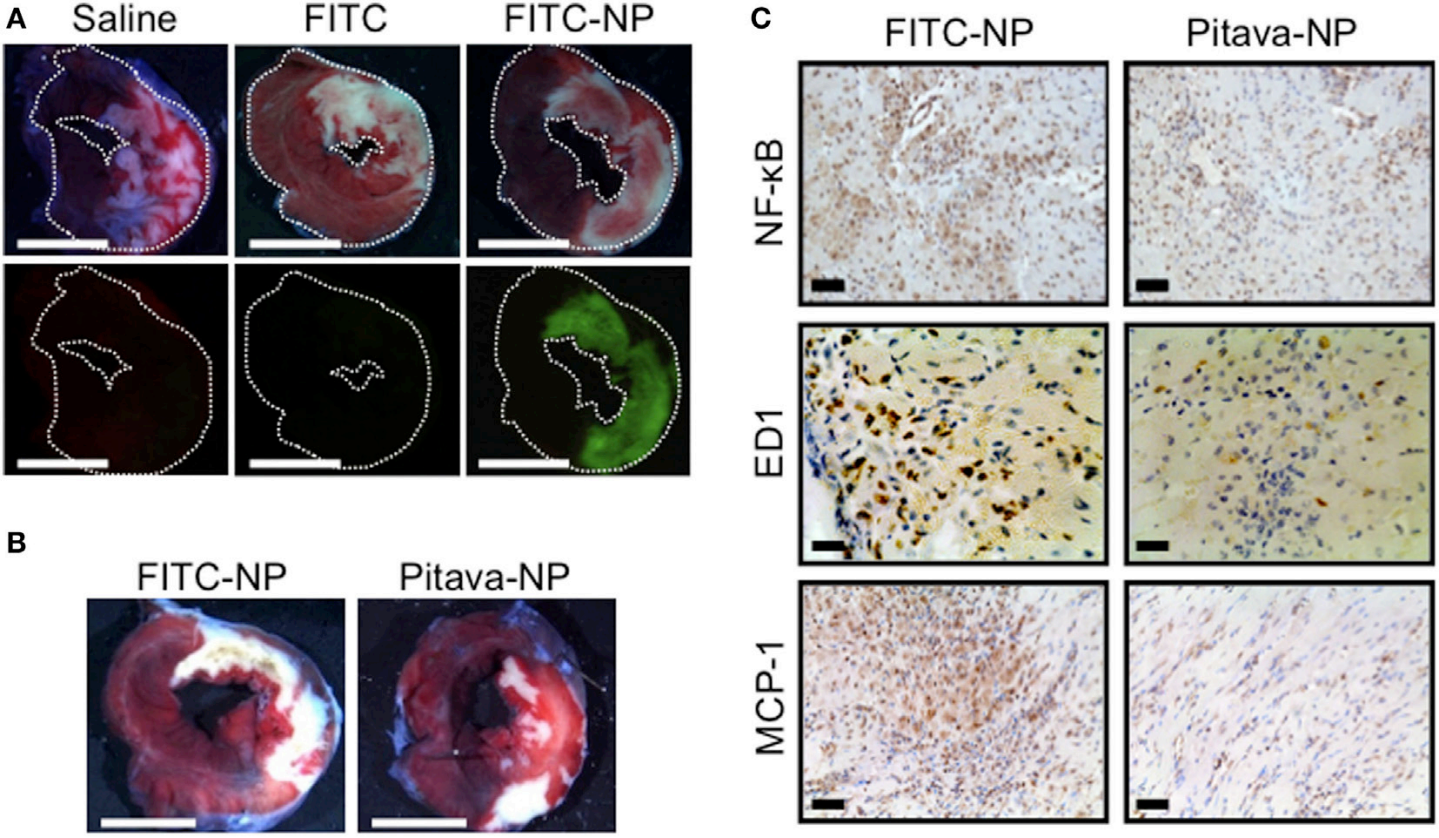
Efficacy of pitavastatin-nanoparticle in myocardial ischemia-reperfusion injury. **(A)** Representative light (upper) and fluorescence (lower) stereomicrographs of cross-section of the ischemia-reperfusion (IR) hearts 3 h after intravenous injection of saline, FITC alone, or FITC-NP. **(B)** Intravenous treatment with Pitava-NP at the time of reperfusion reduced infarct size 24 h after reperfusion. **(C)** Cross-sections from IR myocardium stained with NF-κB, ED-1, and monocyte chemoattractant protein-1 (MCP-1). We reused these data according to the Copyright Transfer Agreement with the publisher (69).

Several pharmacological agents, including statins and erythropoietin analogs, have been shown to reduce MI size in preclinical studies (44). However, several clinical trials on pharmacological cardioprotection for myocardial IR injury have failed to demonstrate a positive impact on clinical outcome, and there is no effective therapy for preventing myocardial reperfusion injury in STEMI patients (44, 67). One possible explanation for the failure of current clinical trials is an insufficient drug delivery during a limited interventional time window, while administered at the time of reperfusion. Therefore, from a clinical perspective, it is feasible to apply an effective DDS that facilitates delivery to the sites of IR injury during reperfusion, a clinically feasible time point. In addition to macrophage-mediated inflammation, the activation of pro-survival kinases including PI3K/Akt and Erk1/2 that are known as reperfusion injury salvage kinases (RISK) in ischemic myocardium is another potential therapeutic target to reduce reperfusion-induced necrosis and apoptosis, and then limits MI size. Statins are known to afford cardioprotection from IR injury in animals (68). The cardioprotection of statins on infarct size is mediated partly by activating the RISK pathway. Intravenously injected nanoparticles accumulate not only in MPS but also in injured tissues, including IR myocardium, where vascular permeability is enhanced (13). Thus, nano-DDS may be a feasible for myocardial IR injury targeting both inflammatory monocytes/macrophages and ischemic myocardium. Therefore, we examined the efficacy of Pitava-NP as a DDS for myocardial IR injury. In a rat model of myocardial 30-min IR, PLGA nanoparticles were found exclusively in the ischemic myocardium (Figure 3A). Flow cytometric analysis showed the incorporation of FITC-NPs in CD11b^+^ leukocytes in blood and IR heart. Intravenous treatment with Pitava-NPs at the time of myocardial reperfusion significantly reduced infarct size 24 h after reperfusion (Figure 3B). The therapeutic effect of Pitava-NP derived from the inhibition of MCP-1/CCR2 pathway by inactivation of NF-κB, which resulted in reduced macrophage-mediated inflammation (Figure 3C) and the activation of RISK pathway in ischemic myocardium (69). To establish preclinical proof-of-concept, we also demonstrated the therapeutic efficiency of Pitava-NPs in a preclinical conscious pig IR injury model (70). These data suggested that nano-DDS of statin to inflammatory cells and ischemic myocardium is a feasible strategy for the treatment of myocardial IR injury. Other nanomaterials can be applied as a nano-DDS for myocardial IR injury. PEGylated liposomes also showed the prolonged circulating time in blood and the specific accumulation in ischemic/reperfused myocardium. The liposomes encapsulating adenosine demonstrated the enhanced cardioprotection effects of adenosine against myocardial IR injury in rats (71). Dendrimer nanoparticles might be another candidate for the nano-DDS due to selective localization in ischemic myocardium (72). Further studies are needed to assess the therapeutic effects of a dendrimer–drug conjugate in myocardial IR injury.

### Post-MI LV Remodeling

**Figure 4:**
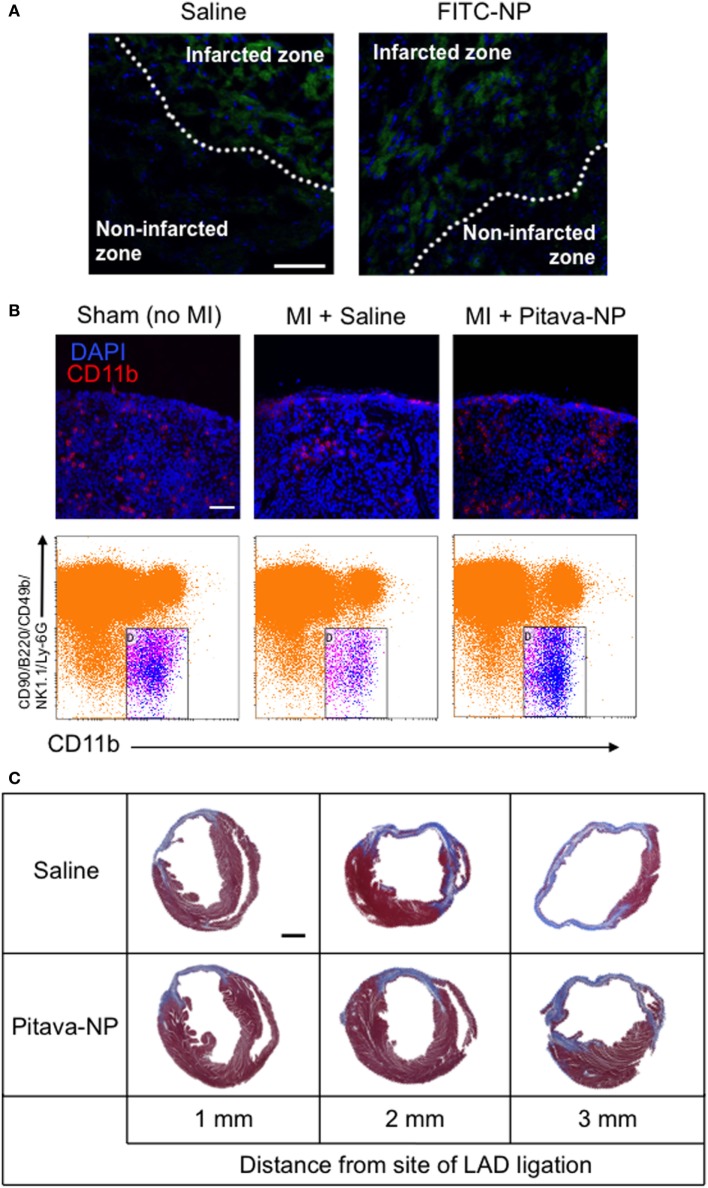
Efficacy of pitavastatin-nanoparticle in post-myocardial infarction left ventricular (LV) remodeling. **(A)** Cross sectional pictures of the infarcted myocardium observed under fluorescent microscope 3 days after LAD ligation. Saline or FITC-NP were injected for three consecutive days after ligation. Weak fluorescent signals observed in infarcted zones are auto-fluorescence from dead cardiomyocytes. **(B)** (Upper panel) Splenic sections stained with anti-CD11b antibody (red) and DAPI (blue) showing the subcapsular red pulp. (Lower panel) Dot plots of flow cytometric analysis of spleen samples. Treatment with Pitava-NPs inhibited MI-induced Lineage (CD90/B220/CD49b/NK1.1/Ly-6G)^−^CD11b^+^ monocyte reduction in the spleen. **(C)** Intravenous treatment with Pitava-NPs for three consecutive days ameliorated LV remodeling 4 weeks after myocardial infarction. We reused these data according to the Copyright Transfer Agreement with the publisher (76).

Recent advances in therapeutic strategy including coronary intervention and optimal medication decreased the acute phase mortality of MI, but the prevalence of chronic heart failure in MI survivors is increasing (73). Intractable heart failure due to LV remodeling (dilatation and remodeling) associated with increased fibrosis and wall thinning after MI remains a major clinical concern. Therefore, there is also an unmet need for cardioprotective modalities to ameliorate post-infarct LV remodeling. The infarcts show high inflammatory response with the recruitment of innate immune cells including macrophages and monocytes derived from extramedullary hematopoiesis such as spleen (74). We have examined the efficacy of PLGA nanoparticles as a DDS for post-infarct LV remodeling. After permanent left anterior descending coronary artery (LAD) ligation, Pitava-NP was intravenously administered for three consecutive days. Intravenously injected FITC-NPs were delivered to Lineage^−^CD11b^+^ monocytes in the blood, spleen, and heart, but not in non-infarcted myocardium within the area at risk due to coronary ligation (Figure 4A), suggesting that the beneficial effects of Pitava-NPs on post-infarct LV remodeling can be predominantly derived from those on inflammatory cells. Actually, Pitava-NP decreased macrophage accumulation in the heart by inhibiting not only angiotensin II-mediated monocyte mobilization from the spleen (Figure 4B) but also monocyte mobilization from the bone marrow that is supposed to be mediated by CCR2 (75). Intravenous treatment with Pitava-NPs after MI induced by permanent LAD ligation attenuated post-infarct LV remodeling associated with reduced monocyte/macrophage accumulation in the heart (Figure 4C) (76). A delayed transition from inflammatory to anti-inflammatory macrophages by prolonged recruitment of classical inflammatory monocytes to myocardium may also interfere with post-infarct LV remodeling (77). Other investigators have intervened a transcription factor that shifts macrophages to inflammatory subsets by utilizing siRNA lipid nanoparticles targeting interferon regulatory factor 5 (IRF5). They demonstrated that siIRF5 improves post-infarct LV remodeling by reprogramming macrophages toward anti-inflammatory subsets (78).

## Summary and Clinical Perspective

In this review, we have summarized a series of evidence including (1) the anti-inflammatory therapeutics for coronary artery disease, (2) a variety of nanomaterials for drug delivery systems, (3) the role and imaging of monocytes/macrophages in coronary artery disease, and (4) application of nano-DDS based on PLGA nanoparticle to coronary artery disease, such as plaque destabilization, IR injury, and post-MI LV remodeling. Applications of nano-DDS for coronary artery disease is a feasible strategy by utilizing increased incorporation of nanomaterials by MPS and enhanced permeability of inflammatory vasculature (Figure 1). In addition to the therapeutic effects of nano-DDS on coronary artery disease, there are a wide variety of opportunities to combine nano-DDS and various therapeutic agents, including chemical, nucleotide, peptide, and others, which may extend the possibility of current pharmacotherapy for several cardiovascular diseases. Possible application of nano-DDS in other cardiovascular diseases may include, pulmonary hypertension (79, 80), vein graft disease (81), and coronary artery stents (82). The clinical application of nano-DDS utilizing Good Manufacturing Practice-compliant Pitava-NPs in this review is in progress. We have already completed phase I/IIa investigators’ initiated clinical trial testing the efficacy of Pitava-NP in patients with critical limb ischemia in the Kyushu University Hospital (UMIN000008011). We have also completed a phase I clinical trial testing the safety of an intravenous administration of Pitava-NP in healthy volunteers (UMIN000014940). Recently, phase III CANTOS trial targeting interleukin-1β with human anti-IL-1β monoclonal antibody, canakinumab, has demonstrated that the inhibition of systemic inflammation may reduce cardiovascular risk even in the absence of concomitant lipid lowering (83). This trial has proved the concept that anti-inflammatory therapeutics targeting innate immunity pathway can be applicable in humans, and our novel DDS utilizing PLGA polymer-based nanotechnology might be one of promising therapeutic strategies for various cardiovascular diseases in the near future.

## Author Contributions

SK, TM, J-iK, KN, and KE make substantial contributions to conception and design and acquisition, analysis, and interpretation of data.

## Conflict of Interest Statement

KE is the inventor of an issued patent on part of the results reported in the present study (Pharmaceutical composition containing statin-encapsulated nanoparticle, WO 2008/026702). Applicants for this patent include Kyushu University (https://airimaq.kyushu-u.ac.jp/), KOWA Inc. (http://www.kowa.co.jp), and Sentan Medical Inc. (http://sentaniryou.co.jp). Sentan Medical Inc. is a drug discovery venture company from Kyushu University. KE is a founder of Sentan Medical Inc., possesses stocks, serves as one of Directors of the company, and reports personal fees from the company outside the submitted work. The intellectual property division of Kyushu University is reviewing that Sentan Medical Inc. did not play a direct role in the study design, data collection and analysis, decision to publish, or preparation of the manuscript in KE’s Laboratory.
